# Hair Dyes

**Published:** 1856-04

**Authors:** 


### HAIR DYES.

One of the European journals relates the case of a gentleman who became a maniac in consequence, as said, of the free use of a hair dye. We know of no efficient hair dye which does not owe its prompt virtues to a solution of "nitrate of silver," which in its solid state is known by the name of "*Lunar Caustic*;" it stains the skin black, by burning it, and will burn into the flesh, if steadily applied. A hot iron will sear the skin, and render it hard, callous, unfeeling, and unfit for natural purposes, preventing that free evaporation, which is essential to the health of the body. If this is done by investing a man with an India Rubber garment, he will die in a few hours.

Hair dyes for whiskers have become very common of late years, they have to be repeated once a month, their more immediate effect is to impart a dead, black color, which at once reveals the hypocrisy, and that it should so disturb the natural functions of the skin, by such frequent application, as to lay the foundation for callosities, cancers, and other affections, is at least to be apprehended. The employment of such cheateries is altogether incompatible with that feeling of independence and self-respect which characterizes an educated gentleman.

